# *The Organon* and Materia Medica Club of the Bay Cities of California

**Published:** 1897-06

**Authors:** W. E. Ledyard

**Affiliations:** M. D.; B. A., M. B., M. R. C. S. Eng., Secretary


### *THE ORGANON* AND MATERIA MEDICA CLUB OF THE BAY CITIES OF CALIFORNIA.

The regular semi-monthly meeting was held at the office of Dr. J. M. Selfridge, 1068 Broadway, corner of Twelfth Street, Oakland, Friday evening, February 5th, 1897.

The reading of the minutes of the last meeting was omitted.

Members present: Drs. J. M. and C. M. Selfridge, Augur, and Ledyard.

The meeting was called to order at 8.20 p. m., by the President, Dr. J. M. Selfridge.

Dr. J. M. Selfridge proposed for senior membership Dr. Charles Lathrop Dyer, of North Berkeley. The proposition was referred to the Board of Censors.

Sections 259 to 283 of *The Organon* were read and discussed.

Referring to the note to Section 260 two of the members present stated that they did not adhere to such strict rules of diet in treating the sick.

Dr. J. M. Selfridge, referring to Section 263, said he would not defer to the wishes of the patient in certain cases when the latter desired to be uncovered, thinking the patient might "take cold."

Dr. J. M. Selfridge, in discussing Section 274, mentioned a case in which he was called in consultation. *Tartar emetic* was clearly the indicated remedy. The husband believed in Homeopathy. The latter was giving drugs by inhalation, flax-seed tea, etc., and the highly potentized medicine at the same time.

Dr. Ledyard—They don't have faith in the medicine, and accordingly think they have to bolster it up.

Dr. C. M. Selfridge—They give the medicine too low, as referred to in Section 275.

Dr. J. M. Selfridge, referring to Section 276, said: "Take a remedy capable of producing a depressing or stimulating effect, *e. g.*, *Camphor*, which is a depressing medicine; it slows up the molecular action of the system, while *Aconite*, which is stimulating, increases its molecular action.

Dr. Ledyard—Nux-vomica sometimes gives rise to terrible aggravations. Because of its depressing effect, there are certain nervous persons in whom it always aggravates.

Dr. C. M. Selfridge has had families in which Bry. and Bell. produced most intense aggravations.

Dr. Ledyard has seen this result follow the administration of Sulph., Phos., Lyc., Bell., etc.

Dr. J. M. Selfridge, referring to note to Section 246, said that he was absolutely certain that in many cases in which the 200th failed to cure the CM. cured. He mentioned a Rhus case, in which the 200th relieved, but didn't cure; the CM., however, cured.

Dr. Ledyard referred to an *Ars.* case, with agonizing pains in the abdomen. *Ars.* 200 at first relieved speedily, but on a return of the pains failed to give any relief. In this case the 40m. potency of the same medicine "acted like a charm," and its administration was followed by speedy recovery.

Dr. Augur, in discussing Section 279, remarked that he was under the impression that if a melicine produces a short, sharp aggravation it is the correct remedy, but if the aggravation continues a long time the medicine has been incorrectly chosen. He mentioned a case in which Sulphur produced an intense and long-continued aggravation. On re-studying the case he gave one dose of *Psorinum* 40m., with speedy relief.

Dr. C. M. Selfridge alluded to a case of eruption, in which the aggravation that followed the administration of Sulphur was so great that, although the eruption disappeared, the patient died.

Dr. Augur, discussing Section 283, remembered that Dr. Biegler said that in treating chronic diseases he would rather give a lower potency as an intercurrent, on account of the severe aggravation following the exhibition of the high potency.

On motion the Club adjourned to meet on the third Friday of the month, at the office of Dr. G. H. Martin, 606 Sutter Street, San Francisco.

W. E. Ledyard, B. A., M. B., M. R. C. S. Eng.,
*Secretary.*

